# Commentary: Older adults can improve compensatory stepping with repeated postural perturbations

**DOI:** 10.3389/fnagi.2016.00111

**Published:** 2016-05-10

**Authors:** Christopher McCrum, Johannes M. N. Essers, Li-Juan Jie, Wai-Yan Liu, Kenneth Meijer

**Affiliations:** ^1^Department of Human Movement Science, NUTRIM School of Nutrition and Translational Research in Metabolism, Maastricht University Medical Centre+Maastricht, Netherlands; ^2^Institute of Movement and Sport Gerontology, German Sport University CologneCologne, Germany; ^3^Faculty of Health, Research Centre for Autonomy and Participation of Persons with a Chronic Illness, Zuyd University of Applied SciencesHeerlen, Netherlands; ^4^Department of Family Medicine, CAPHRI School for Public Health and Primary Care, Maastricht University Medical Centre+Maastricht, Netherlands; ^5^CIRO, Department of Research and EducationHorn, Netherlands

**Keywords:** age, dynamic stability, motor control, adaptation potential, falls, balance, locomotion

Falls in older people often occur during ambulation, frequently due to trips or slips (Berg et al., 1997), and are associated with many negative health outcomes (Terroso et al., 2014). Consequently, adaptation and improvement of compensatory stepping responses (which are less effective in old age; Maki and Mcilroy, 2006) to mechanical perturbations, could reduce falls risk in older people. As involuntary stepping responses to unexpected perturbations are faster than voluntary steps (Luchies et al., 1999), unexpected perturbations may be the most appropriate stimulus for improving stepping responses (Oddsson et al., 2007).

In their recent paper, Dijkstra et al. (2015) examined young and older adults' improvement, retention, and inter-task transfer in compensatory stepping responses to stance perturbations. The authors determined the center of mass displacement, stepping behavior, and lower limb muscle activation in young and older adults during multi-direction surface translations in two sessions separated by 24 h. The authors concluded that older adults were capable of reducing their center of mass displacement with task repetition (indicating improved stability control), but improvements in recovery stepping responses in the anteroposterior direction did not transfer to the mediolateral direction. This is a key finding, as it indicates the importance of multi-directional stepping training for older adults.

While the authors focused their discussion on perturbations during stance and sit-to-stand situations, other studies have demonstrated that older adults can successfully adapt to perturbations during gait. In particular, Bierbaum et al. (2010, 2011) found similar results to Dijkstra et al. (2015) using surface compliance changes during gait. These studies found that older adults were capable of favorable reactive and predictive adaptations in gait that were, in some cases, of a greater magnitude to those of young adults, indicating the presence of floor effects. Repeated slips during gait have also led to favorable adaptations in older adults' recovery responses (Bhatt et al., 2012; Pai et al., 2014). Additionally, a recent meta-analysis concluded that both reactive and predictive locomotor adaptations show only minor, non-significant declines with age (Bohm et al., 2015). The above mentioned studies, together with the work in stance (Mansfield et al., 2010) and sit-to-stand (Pavol et al., 2002, 2004) discussed by Dijkstra et al. (2015), indicate that, with sufficient practice, healthy older adults are capable of adapting and improving their postural compensation to a range of stance and gait perturbations.

Despite the study's findings aligning well with previous results, a few issues should be highlighted. While the authors aimed to control for startle responses by conducting familiarization trials (named “first perturbations” in the paper), they may have excluded some relevant between-group differences by not including the familiarization trials, and hence, startle responses in the analysis which may have influenced the conclusions (Allum et al., 2011). For example, Dijkstra et al. (2015) identified similar rates of improvement between the age groups for forward stepping but this only accounted for the motor practice block, and ignored these early perturbations. During these seven anteroposterior perturbations (mediolateral perturbations did not transfer), the older adults may have not performed and adapted as well or as quickly as the young adults. Our group recently observed such results in a study of young, middle and older aged adults' gait in which we applied a sustained resistance perturbation for 18 steps. We found that the older adults' first three steps during the perturbation were significantly less stable compared to the other groups' first three steps, despite them adapting and reaching comparable stability to the younger groups with further repetition (McCrum et al., 2016).

A second issue relates to the retention measurements, as these same “first perturbations” were applied before the perturbations used to determine retention. Here, in addition to potential age related differences, acute adaptations to these lower intensity perturbations may have positively influenced performance during the stronger perturbations (Patel and Bhatt, 2015), potentially resulting in an overestimation of retention. However, these first perturbations were required to determine each individual's stepping threshold using three different intensities in each direction. Despite this approach, some floor effects were still found in the younger adults.

The authors identified the 24 h between measurements as a limitation in the study design. Previous studies have found that older adults retain some beneficial adaptations of stability control in response to one session of repeated slipping perturbations for up to 6 (Bhatt et al., 2012; Pai et al., 2014), 9, and 12 months (Pai et al., 2014). Therefore, the results of Dijkstra et al. (2015) could be expected and are in line with previous results, despite the possibility of an overestimation of retention. Furthermore, these data could be used as a reference group for comparison with patient groups with severely affected locomotor abilities (stroke or Parkinson's disease, for example), in order to examine deficits in short term retention in balance and locomotor tasks.

In sum, the results of Dijkstra et al. (2015) are of importance and significance for the field of falls prevention and stability control in aging. In particular, the work highlights the importance of multidirectional step or perturbation training, due to a lack of transfer across tasks. Whether this would hold for multidirectional gait perturbations is unclear, due to the influence of forward velocity during walking. Future work should explore different types, intensities and frequencies of perturbations in order to determine the most effective strategy for improving dynamic stability control in healthy older adults and in patients with declined locomotor performance and increased falls risk. Finally, as Dijkstra et al. (2015) and previous studies found floor effects in the adaptation of young participants, further attempts should be made to appropriately scale perturbations to participant or group ability, in order to reliably compare adaptation across different groups.

## Author contributions

All authors were involved in the analysis, discussion, and critical revision for important intellectual content related to this manuscript. CM conceived the idea for this work and drafted the manuscript.

## Funding

CM was funded by the Kootstra Talent Fellowship awarded by the Centre for Research Innovation, Support and Policy (CRISP) of Maastricht University Medical Center and by the NUTRIM School of Nutrition and Translational Research in Metabolism NWO Graduate Programme financially supported by the Netherlands Organisation for Scientific Research.

### Conflict of interest statement

The authors declare that the research was conducted in the absence of any commercial or financial relationships that could be construed as a potential conflict of interest.
